# Ribosomal protection as a linezolid resistance mechanism in *Mycobacterium abscessus*

**DOI:** 10.1128/aac.01605-25

**Published:** 2026-03-02

**Authors:** Tobias Funck, Kerry McGowen, Mark R. Sullivan, Samuel Zinga, Ian D. Wolf, Dennis Nurjadi, Claudia M. Denkinger, Eric J. Rubin

**Affiliations:** 1 Department of Immunology and Infectious Disease, Harvard T.H. Chan School of Public Health 1857 , Boston, Massachusetts, USA; 2 Department of Infectious Disease and Tropical Medicine, Heidelberg University Hospital & German Center of Infection Research partner site, Heidelberg, Germany; 3 Institute of Medical Microbiology, University of Lübeck & University Hospital Schleswig-Holstein Campus Lübeck, Lübeck, Germany; Queen Mary University of London, London, United Kingdom

**Keywords:** *Mycobacterium abscessus*, ribosomal protection, mycobacteria, ABC-F proteins, antibiotic resistance, antibiotics

## Abstract

*Mycobacterium abscessus* has emerged as a significant pulmonary pathogen characterized by its resistance to most first-line antimycobacterial drugs. Recent investigations have highlighted the clinical efficacy of including the oxazolidinone antibiotic linezolid in *M. abscessus* combination therapies, despite moderate resistance frequently being observed in patient isolates. Even with the potential usefulness of linezolid, the mechanisms that drive linezolid resistance in *M. abscessus* remain poorly understood. In several bacterial pathogens, including *Mycobacterium tuberculosis*, ATP-binding cassette (ABC) family proteins of the F subtype (ABC-F) have been found to confer antibiotic resistance to ribosome-targeting antibiotics, including linezolid. Here, we identified an *M. abscessus* ABC-F protein, MAB_2736c, that causes specific resistance to antibiotics that bind the 50S ribosomal subunit, including linezolid, macrolides, and chloramphenicol. These results demonstrate that targeting ABC-F proteins could help combat intrinsic resistance to several ribosome-targeting antibiotics in mycobacteria.

## INTRODUCTION

*Mycobacterium abscessus* is one of the few nontuberculous mycobacteria (NTM) species that can cause clinically significant infections in humans and is one of the pathogens responsible for the rise in opportunistic lung infections over the past two decades (1). *M. abscessus* infection is notoriously difficult to treat, with cure rates estimated at less than 50% (2, 3), primarily due to broad-spectrum antibiotic resistance (4). Consequently, current treatment regimens for *M. abscessus* infections typically require combination therapies lasting 18−24 months (5). This highlights the need for improved therapeutic options. The oxazolidinone antibiotic linezolid was recently shown to improve clinical outcomes when included in regimens to treat *M. abscessus* infections (6, 7). Additionally, linezolid substantially improved treatment success when included in multidrug regimens to treat tuberculosis, a disease caused by a closely related pathogen, *Mycobacterium tuberculosis* (Mtb). Linezolid is now included in the World Health Organization guidelines for multidrug-resistant tuberculosis (8, 9). Since *M. abscessus* has limited therapeutic options and linezolid has conceivable potential to be included in regimens, it is imperative to better understand linezolid resistance in *M. abscessus*.

There are numerous routes that bacteria can take to achieve drug resistance. An increasingly recognized mechanism of resistance to ribosome-binding antibiotics involves F subtype ATP-binding cassette (ABC-F) proteins. The ABC superfamily is an ancient group of diverse proteins ubiquitous across all kingdoms of life. ATP-dependent transporters are the most well-known and well-characterized ABC family proteins, with many implicated in multidrug efflux (10, 11). However, several proteins involved in drug resistance containing an ABC cassette have recently been re-characterized from membrane transporters to ribosome-binding proteins. These ABC-F family proteins are located in the cytosol, where they bind directly to ribosomes and modulate their function, instead of transporting molecules across membranes (12, 13). Consequently, several ABC-F family proteins confer antibiotic resistance (ARE) by binding the ribosome to alleviate translational inhibition from antibiotics that target the large ribosomal subunit (12–17).

ARE-ABC-F proteins are categorized into three functional groups based on the antibiotics to which they confer resistance: (i) PLS_A_ for those that protect against pleuromutilins, lincosamides, and streptogramins A; (ii) MS_B_ for those that protect against macrolides and streptogramins B; and (iii) PhO for those that protect from phenicols and oxazolidinones (12–17). All ARE-ABC-F proteins share two similar ABC nucleotide-binding domains that are connected by a helical linker called the antibiotic-resistance domain (ARD). The ARD has been shown to interact with the P-site tRNA in several ARE-ABC-F proteins. This allows the ARE-ABC-F protein to access the peptidyl transferase center, leading to a cascade of conformational changes that result in the displacement of antibiotics from their binding sites (14, 18–20).

PLS_A_ and MS_B_ ARE-ABC-F proteins have been well-characterized across bacterial species, including *M. abscessus* (21); however, PhO ARE-ABC-F proteins are less well-studied. Several PhO ARE-ABC-F proteins have recently been identified and characterized in other pathogenic species. For example, OptrA (22–25) and PoxtA (14, 26, 27), found in *Enterococcus* and *Staphylococcus* species, have been shown to be important antibiotic resistance factors with significant clinical implications. Furthermore, crystal structures of these proteins have revealed structural evidence for the ability of PhO ARE-ABC-F proteins to dislodge oxazolidinones and phenicols (14). Additionally, recent work has demonstrated that OcrA (Rv1473) in Mtb is also an ARE-ABC-F PhO protein that confers resistance to linezolid (28). These emerging pieces of evidence led us to investigate whether *M. abscessus* linezolid resistance is driven in part by the action of ABC-F family proteins.

## RESULTS

### *MAB_2736c* likely encodes for an antibiotic resistance ABC-F

**Fig 1:**
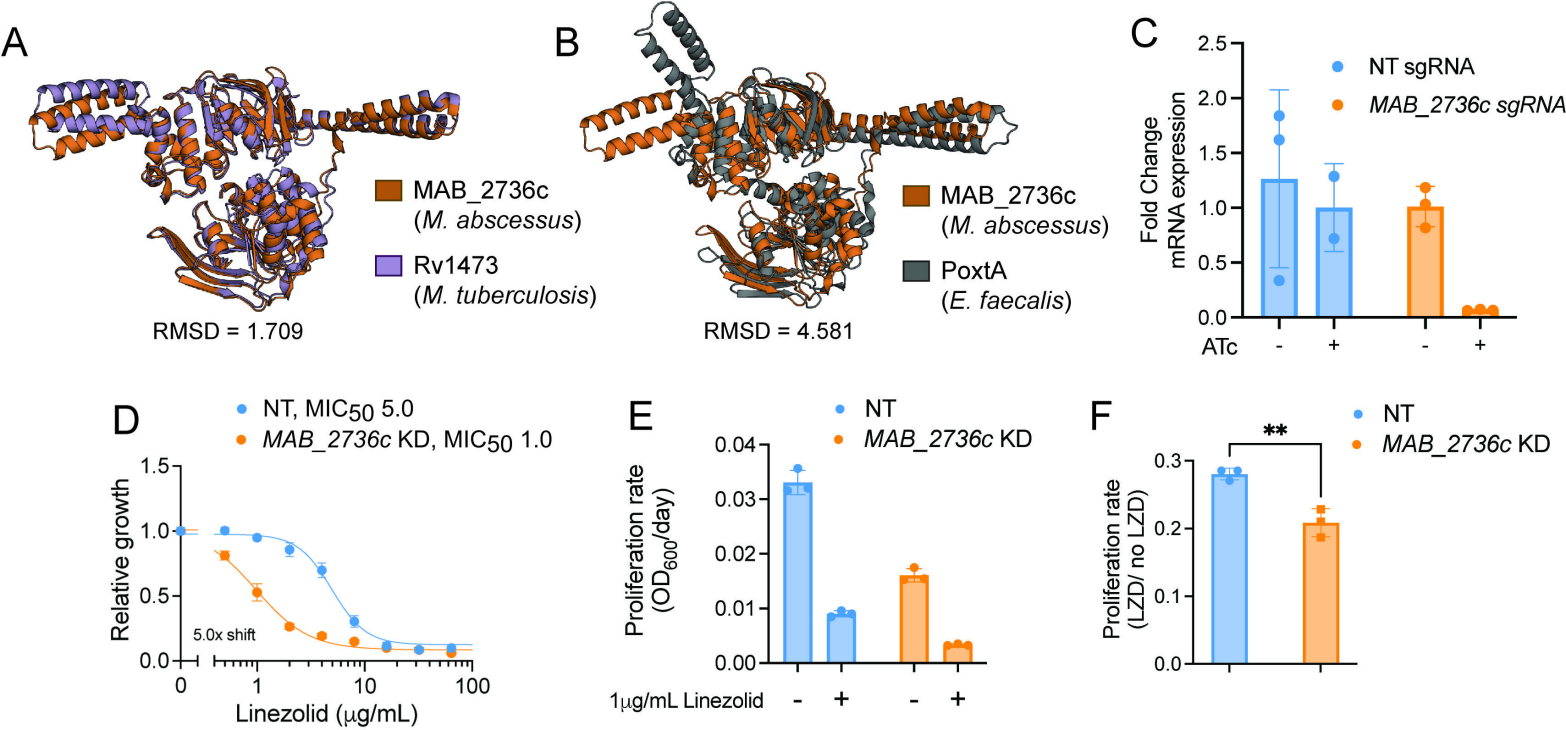
MAB_2736c knockdown displays increased sensitivity to linezolid. (**A**) Alignment of predicted protein structure of MAB_2736c from *M. abscessus* ATCC 19,977 (orange) with predicted protein structure of Rv1473 from *M. tuberculosis* H37Rv (purple), and (**B**) PoxtA from *E. faecalis* crystal structure (gray). R.M.S.D = root mean square deviation. (**C**) Fold change mRNA expression of non-targeting (NT) sgRNA and *MAB_2736c* sgRNA in the presence or absence of 500 ng mL^−1^ ATc. (**D**) Relative proliferation as measured by resazurin reduction for *M. abscessus* ATCC19977 with CRISPRi construct targeting *MAB_2736c* or NT treated with indicated concentrations of linezolid in the presence of 500 ng mL^−1^ ATc. Values normalized to DMSO vehicle. MIC_50_ in µg/mL. (**E**) Proliferation rates calculated from OD600 measurements for an *MAB_2736c* knockdown (KD) and a non-targeting control (NT) in the presence of 500 ng mL^−1^ ATc in *M. abscessus* ATCC19977 grown in the indicated concentration of linezolid for 48 h. Values normalized to ATc-uninduced cells. (**F**) Ratio of proliferation rates under linezolid treatment normalized to vehicle treatment. *P*-value derived from an unpaired, two-tailed *t*-test. For (**C–F**), *n* = 3 biological replicates, and data are presented as individual values along with mean ± s.d. NT, non-targeting; KD, knockdown; ATc, anhydrous tetracycline; LZD, linezolid.

A homology search of amino acid sequences revealed that the *M. abscessus* gene *MAB_2736c* shares 84% sequence identity with that of *ocrA* (*Rv1473*) from Mtb (29, 30). To validate that *MAB_2736c* encodes an ABC-F family protein, its predicted tertiary structure was generated using AlphaFold (31), and this predicted structure was aligned with the predicted structure of OcrA, as well as the X-ray crystal structure of PoxtA from *Enterococcus faecalis* (14) (Fig. 1A and B). Structural alignment of MAB_2736c revealed a root mean square deviation (RMSD) of 1.709 Å relative to OcrA, supporting its classification as an ABC-F family protein. Although the RMSD relative to PoxtA is higher (4.581 Å), the two proteins share visually similar folds and conserved structural motifs (Fig. 1A and B), further supporting a functional relationship despite the overall lower structural alignment.

We next sought to determine whether MAB_2736c might play a role in linezolid resistance. We utilized an anhydrotetracycline (ATc)-inducible Cas9/CRISPRi system in the *M. abscessus* subspecies *abscessus* reference strain (ATC19977) to determine whether depletion of this protein is sufficient to modulate linezolid susceptibility (32, 33). We found that the induced knockdown of *MAB_2736c* (Fig. 1C) exhibits an increased sensitivity to linezolid (Fig. 1D through F). Together, these data suggest that MAB_2736c is most likely an ARE-ABC-F protein and could play a role in antibiotic resistance similar to OcrA and other PhO proteins.

### Overexpression of *MAB_2736c* increases linezolid resistance

**Fig 2:**
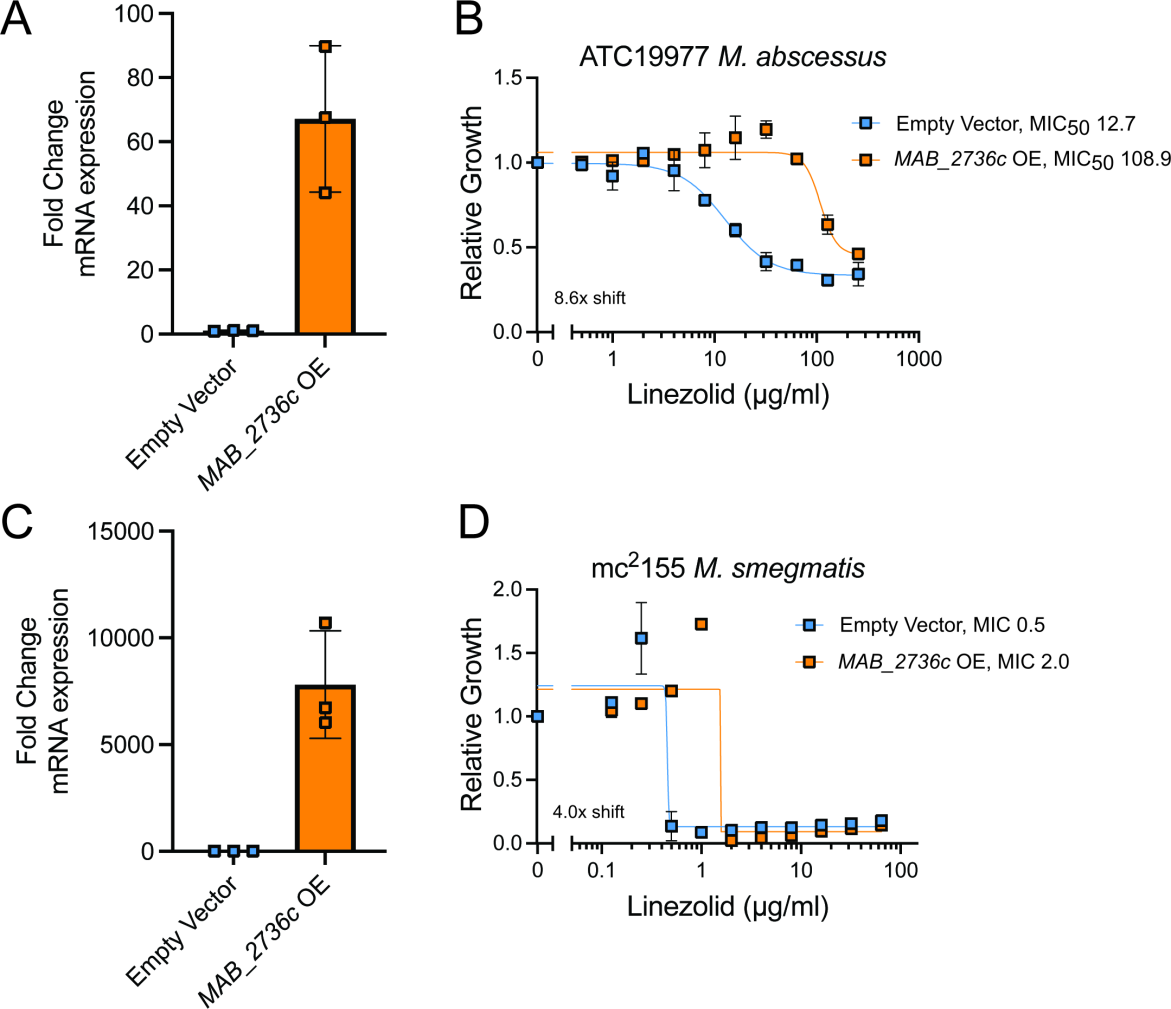
MAB_2736c overexpression is sufficient to cause linezolid resistance. Fold change mRNA expression of empty vector control and *MAB_2736c* overexpressing (OE) vector in (**A**) ATC19977 *M. abscessus* and (**C**) mc^2^155 *M. smegmatis*. Relative proliferation as measured by resazurin reduction for parental strains with an empty vector or strains overexpressing MAB_2736c vector (*MAB_2736c* OE) of (**B**) *M. abscessus* ATCC19977, and (**D**) *M. smegmatis* mc^2^155 exposed to the indicated concentrations of linezolid. Data are presented as individual values along with mean +/− SD. *n* = 3 biological replicates. MIC_50_, MIC in µg/mL. MIC for (**D**) was determined visually as described in the methods.

Although these results indicate that loss of MAB_2736c renders *M. abscessus* more susceptible to linezolid, those effects could be due to indirect translational defects downstream of MAB_2736c knockdown, as other ABC-F family proteins are known to modulate translation in a broad fashion, such as aiding in ribosome assembly (34). To assess whether MAB_2736c can actively modulate linezolid efficacy, we next examined whether *MAB_2736c* overexpression confers increased linezolid resistance. Constitutive overexpression of *MAB_2736c* in the *M. abscessus* reference strain (Fig. 2A) is sufficient to increase levels of resistance to linezolid (Fig. 2B). While this result does not exclude the possibility of indirect translational effects, it is consistent with a direct role in linezolid protection. To determine whether this effect holds broadly true across mycobacteria, we also expressed *MAB_2736c* in *Mycobacterium smegmatis*, a nonpathogenic mycobacterial species. *M. smegmatis* also became significantly more resistant to linezolid with overexpression of *MAB_2736c* (Fig. 2C and D), suggesting that *MAB_2736c* is sufficient to produce linezolid resistance through a mechanism that is generally applicable across mycobacteria.

### Sequence variation does not correlate with linezolid resistance in clinical isolates

To evaluate the clinical relevance of MAB_2736c beyond the type strain, we examined linezolid-resistant *M. abscessus* clinical isolates for sequence variation in ribosomal resistance loci and in the coding and promoter regions of *MAB_2736c*. Nonsynonymous mutations were detected in *rrl* and *rplD* in several isolates. However, these variants did not correspond with resistance levels across the cohort (Table 1). No single ribosomal variant explained the observed phenotypic differences, consistent with prior observations (35). Notably, the variants were subspecies-specific, occurring in *M. abscessus* subsp. *massiliense* isolates but not in subsp. *abscessus*, irrespective of resistance phenotype. We further analyzed the *MAB_2736c* coding sequence and its upstream promoter region (defined as 300 bp upstream of the start codon containing the transcriptional start site). No nonsynonymous sequence variation was observed in the gene. The upstream region of the gene exhibited a high degree of sequence conservation across strains, encompassing the transcriptional start site, the promoter, and a conserved −10 motif (File S1). Within the promoter region, observed polymorphisms did not correlate with linezolid minimum inhibitory concentration (MIC) values but, similar to the ribosomal loci, were specific to subspecies (Table 1). Subspecies-associated sequence divergence has been reported previously in comparative genomic analyses of the *M. abscessus* complex (36, 37). The results from this small sample do not support a role for MAB_2736c coding or promoter polymorphisms as determinants of linezolid resistance in the analyzed clinical isolates and instead suggest that the observed variation reflects subspecies-specific differences.

**TABLE 1:** Sequence variation does not correlate with linezolid resistance in clinical isolates^*a*^

Strain	Subspecies	Linezolid MIC_50_ in µg/mL	Rrl (23S rRNA)	rplC (L3)	rplD (L4)	MAB_2736c	MAB_2736c promoter region
ATCC 19,977	*abscessus*	11.6	WT	WT	WT	WT	WT
BWH-F	*massiliense*	32.3	C634T, T636C, A1717G, T3001C	NNV	WT	NNV	– 20 C>A, – 30 C>A, – 114 A>G, – 219T>G
Taiwan-36	*massiliense*	23.2	C634T, A1717G, T3001C	NNV	Gly144Ala, Ala177Pro	NNV	– 20 C>A, – 30 C>A, – 114 A>G, – 219T>G
Taiwan-38	*massiliense*	26.6	C634T, A1717G, T3001C	NNV	Gly144Ala, Ala177Pro	NNV	– 20 C>A, – 30 C>A, – 114 A>G, – 219T>G
Taiwan-44	*abscessus*	29.4	WT	WT	WT	WT	WT
Taiwan-48	*massiliense*	14.5	C634T, A1717G, T3001C	NNV	Gly144Ala, Ala177Pro	NNV	– 20 C>A, – 30 C>A, – 114 A>G, – 219T>G
Taiwan-54	*massiliense*	19.0	C111T, C634T, A1717G, T3001C	NNV	Gly144Ala	NNV	– 20 C>A, – 30 C>A, – 114 A>G, – 219T>G

^*a*^ 23S rRNA and rplC, rplD, MAB_2736c were analyzed following multiple sequence alignment, protein coding genes examined at the peptide level. WT = wild type, NNV = no nonsynonymous variants. Sequence length differences relative to clinical isolates were observed for rrl (ATCC19977 longer by 6 bp), rplC (ATCC19977 shorter by 36 bp), and rplD (ATCC19977 shorter by 15 bp), MAB_2736c coding sequences were identical in length across all strains. Promoter variants for MAB_2736c are reported extending up to 300 bp upstream of the start codon.

### MAB_2736c changes the susceptibility to multiple ribosome-targeting antibiotics

**Fig 3:**
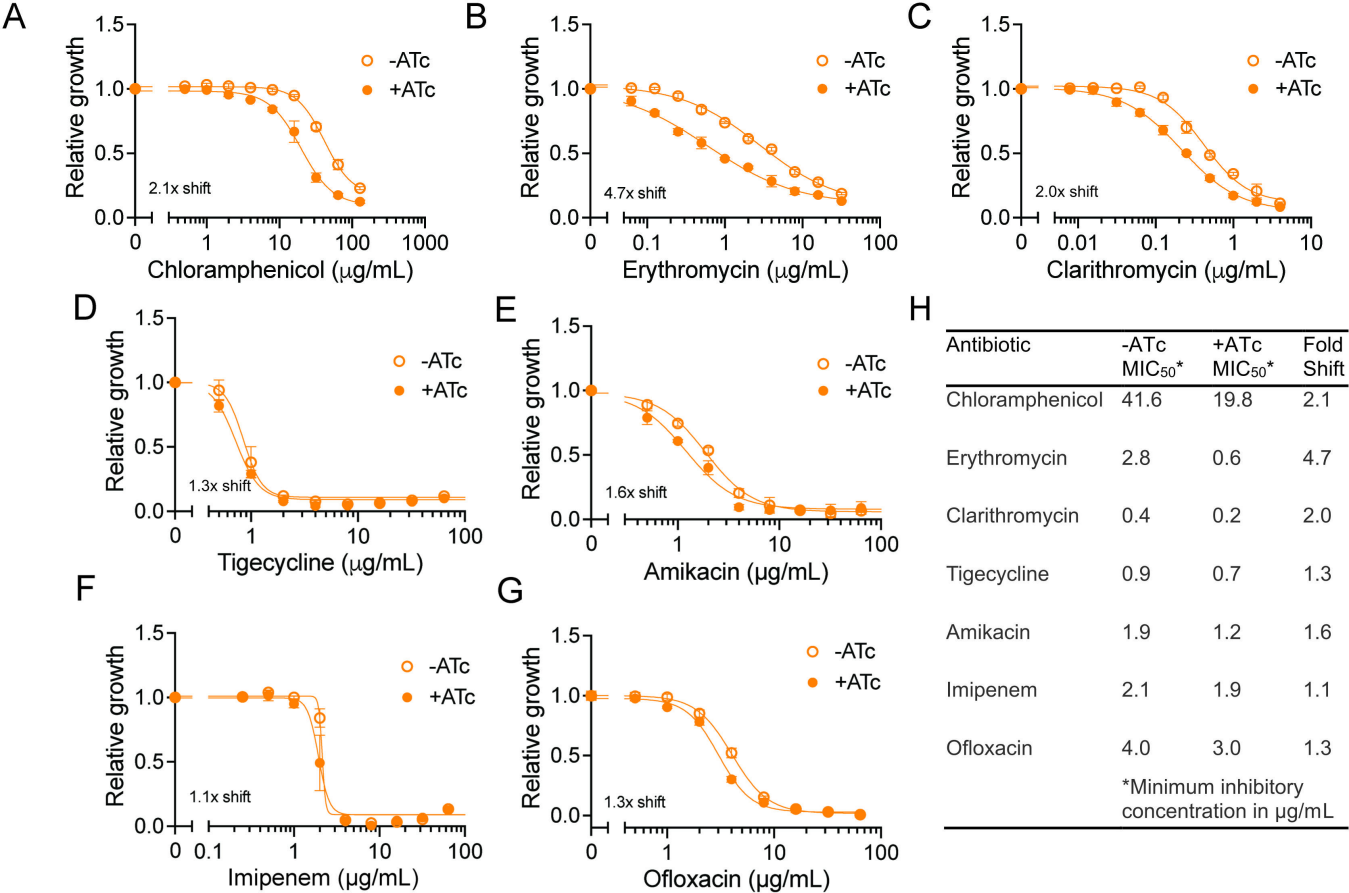
MAB_2736c knockdown mutant is sensitive to multiple 50S ribosome-targeting antibiotics. Relative proliferation as measured by resazurin reduction for *M. abscessus* ATCC19977 with CRISPRi construct targeting *MAB_2736c* treated with indicated concentrations of (**A**) chloramphenicol, (**B**) erythromycin, (**C**) clarithromycin, (**D**) tigecycline, (**E**) amikacin, (**F**) imipenem, and (**G**) ofloxacin in the presence or absence of 500 ng mL^−1^ ATc. Values normalized to DMSO vehicle. *n* = 3 biological replicates. Data are presented as individual values along with mean ± s.d. (**H**) Table summarizing MIC_50_ values derived from panels (**A–G**) under ± ATc conditions. ATc, anhydrous tetracycline.

All previously characterized ARE-ABC-F proteins provide resistance to a certain subset of antibiotics that target the 50S subunit of the ribosome (12–17). Consistent with this observation, *MAB_2736c* knockdown induces increased sensitivity to chloramphenicol (Fig. 3A), a compound whose effect is also modulated by OcrA (28) and other PhO ARE-ABC-F proteins (14, 22–27). Unexpectedly, *MAB_2736c* knockdown also induces sensitivity to the macrolides, erythromycin, and clarithromycin (Fig. 3B and C). This was a notable finding given that other PhO ARE-ABC-F proteins, like PoxtA and OptrA, do not alter sensitivity to macrolides (14). However, earlier work, when OcrA was thought to be an ABC transmembrane drug exporter, showed that *ocrA* (*rv1473*) mutants in Mtb also have an increased susceptibility to macrolides (38). These observations suggest that MAB_2736c may influence resistance more broadly than previously characterized PhO ARE-ABC-F proteins or that MAB_2736c is promoting drug resistance through a different mechanism.

To distinguish these possibilities, we examined whether *MAB_2736c* knockdown affects sensitivity to antibiotics that target translation through the 30S subunit (amikacin and tigecycline) as well as antibiotics that target DNA (ofloxacin) or cell wall synthesis (imipenem), which are not expected to be affected by the action of traditional PhO ARE ABC-F proteins. *MAB_2736c* knockdown does not alter sensitivity to these antibiotics (Fig. 3D through G), suggesting that MAB_2736c provides specific protection against translation inhibitors that target the 50S subunit, similar to other PhO ARE-ABC-F proteins. However, the observed broader range of protection, including macrolides, raises the possibility that MAB_2736c operates through a distinct or partially divergent mechanism compared to other PhO ARE-ABC-F proteins.

### MAB_2736c protects purified ribosomes against linezolid

**Fig 4:**
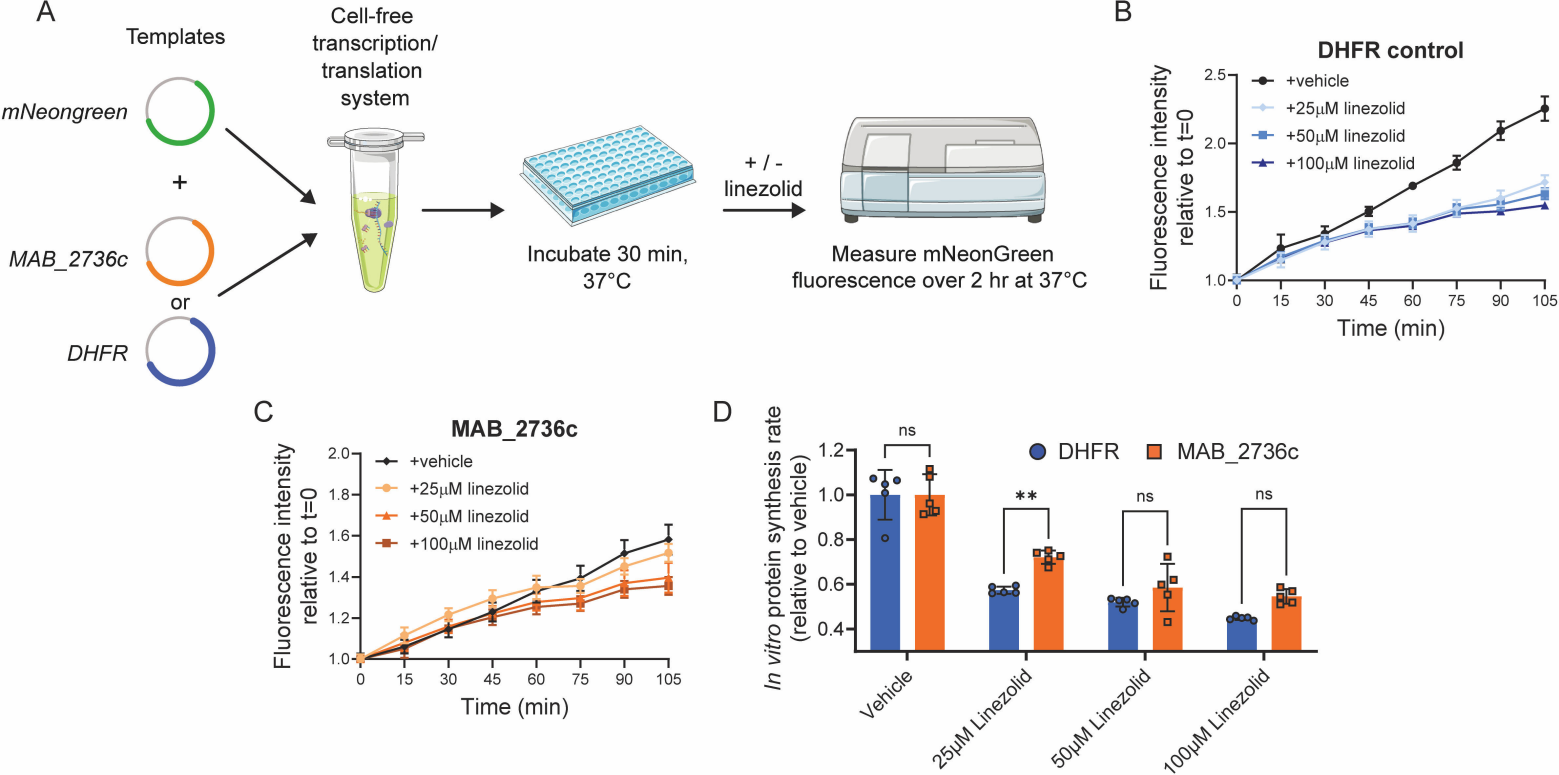
MAB_2736c protects purified ribosomes against linezolid. (**A**) Schematic of cell-free transcription/translation assay derived from *E. coli*. DHFR = Dihydrofolate reductase. (**B**) mNeonGreen fluorescence when co-expressed with DHFR in the presence of 25 μM, 50 μM, and 100 µM linezolid or DMSO. *n* = 5 independent samples. (**C**) mNeonGreen fluorescence when co-expressed with MAB_2736c in the presence of 25 μM, 50 μM, and 100 µM linezolid or DMSO. *n* = 5 independent samples. (**D**) Relative protein synthesis rate for mNeonGreen co-expressed with MAB_2736c or DHFR in the presence of 25 μM, 50 μM, or 100 μM linezolid, or DMSO. Rates were calculated from post-linezolid addition until 105 min elapsed. Data are presented as individual values along with mean +/− SD. *P*-values derived from Šídák’s multiple comparisons test after two-way ANOVA. *n* = 5 biological replicates.

To further characterize the mechanistic effects of MAB_2736c expression on ribosome function, we sought to evaluate the effects of MAB_2736c on translation in a cell-free transcription-translation system that utilizes ribosomes derived from *Escherichia coli* (39). We simultaneously expressed a fluorescent reporter protein, mNeonGreen, with either MAB_2736c or a control protein from *E. coli*, dihydrofolate reductase (DHFR) (Fig. 4A). Following 30 min of undisturbed translation of MAB_2736c or DHFR, we then added linezolid and monitored whether MAB_2736c protected translating ribosomes (Fig. 4A). In the absence of drug, MAB_2736c reduces the fluorescent reporter translation baseline rate compared to either DHFR or the no template control (Fig. 4B and C), suggesting that MAB_2736c may modulate or interfere with *E. coli* ribosome function *in vitro*. The addition of linezolid not only decreases the rate of translation as expected in the control template condition but also produces a modest decrease in translation rate in the MAB_2736c expressing condition (Fig. 4B and C). After normalization to vehicle-treated controls (DMSO) to account for baseline differences, MAB_2736c expression was associated with a modest relative preservation of translation at 25 μM linezolid compared to DHFR (Fig. 4D). This protective effect is diminished at higher doses of linezolid (Fig. 4D), consistent with the observation that more saturating doses of linezolid are able to inhibit *M. abscessus* growth.

These results are consistent with the possibility that MAB_2736c can reduce the susceptibility of translating ribosomes to linezolid inhibition via direct ribosomal protection. However, given the reduced baseline translation and the heterologous nature of this assay, alternative interpretations such as partial inhibition of *E. coli* ribosomes by MAB_2736c leading to less linezolid susceptibility cannot be excluded. Accordingly, we interpret these data as supportive evidence that MAB_2736c functions as a candidate ribosome protection factor, contributing to *M. abscessus* linezolid resistance. Additional studies in homologous systems will be required to define its precise mechanism of action in *M. abscessus*.

## DISCUSSION

Numerous ABC-F proteins have been shown to confer antibiotic resistance in several pathogenic species (12–17). However, their roles in pathogenic mycobacteria remain largely unexplored. In this study, we provided genetic, phenotypic, and mechanistic evidence to classify MAB_2736c in *M. abscessus* as a candidate ARE-ABC-F protein that confers resistance to linezolid, chloramphenicol, and macrolides, likely through a ribosome protection mechanism.

Typically, ARE-ABC-F proteins are categorized into functional groupings based on antibiotics to which they confer resistance. For example, ARE-ABC-F proteins that confer resistance to linezolid and chloramphenicol belong to the PhO group of ARE-ABC-F proteins (14). However, in contrast to other PhO ARE-ABC-F proteins, MAB_2736c expression also alters sensitivity to other 50S ribosomal subunit-targeting antibiotics, including macrolides. Our findings suggest that MAB_2736c could act in a mechanistically distinct manner from other PhO proteins. This distinction highlights the need to re-evaluate the current classification system for ARE-ABC-F proteins. Furthermore, we find a bacterial growth deficit caused by MAB_2736c depletion even in the absence of antibiotic pressure, hinting at an integral biological role of MAB_2736c beyond drug resistance. Further investigation is required to discern how MAB_2736c modulates translation under baseline conditions. Structural insights into how MAB_2736c binds to the mycobacterial ribosome could help illuminate how MAB_2736c differs from other ARE-ABC-F family proteins and provide insight into its native functions. Consequently, this could also help inform targeted drug design for MAB_2736c.

Inhibition of MAB_2736c may enhance the efficacy of multiple clinically relevant ribosome-targeting antibiotics. Furthermore, several avenues to overcome the antibiotic resistance caused by ARE-ABC-F proteins have been proposed; these include the development of ribosome-targeting drugs with higher ribosomal affinity to outcompete ABC-F proteins, as well as ABC‐F protein binding site mimics to inhibit ribosome function to stall further translation (17). Given the broad effects of MAB_2736c on the potency of several classes of ribosome-targeting antibiotics, applying these approaches in *M. abscessus* might be a valuable way to improve our ability to target bacterial translation and combat resistance. Analysis of six linezolid-resistant *M. abscessus* clinical isolates did not identify *MAB_2736c* coding or promoter polymorphisms that segregated with linezolid MIC. These findings suggest that, in this small cohort, MAB_2736c-associated linezolid resistance is unlikely to be explained by genetic variation at the locus and may instead involve broader regulatory mechanisms. Although no association between MAB_2736c sequence variation and linezolid resistance was detected, larger studies across diverse clinical strains may reveal rare or lineage-specific variants not captured here. Such analyses were beyond the scope of this work and, together with *in vivo* validation, will be required to fully assess the clinical relevance of targeting MAB_2736c. Overall, this work advances our understanding of ARE-ABC-F proteins in mycobacteria and supports previous notions that ARE-ABC-F proteins may represent attractive drug targets to potentiate the effects of current antibiotics.

## MATERIALS AND METHODS

### Strains

All experiments were performed in the *Mycobacterium abscessus* subspecies *abscessus* type strain (ATCC19977) unless otherwise indicated. The experiments with *Mycobacterium smegmatis* utilized the strain mc^2^155 (ATCC700084). All plasmid construction was performed in *E. coli* DH5α.

### Oligonucleotides

Oligonucleotide details are provided in Table 2.

**TABLE 2:** Oligonucleotides

Oligonucleotide name	Description	Sequence (5’ to 3’)
2736c_1f_qPCR	Forward qPCR primer for MAB_2736c	cgagcggattctggttcaac
2736c_1r_qPCR	Reverse qPCR primer for MAB_2736c	gctcgacgttgtgactgatc
Mab_16S_rRNA_F	Forward qPCR primer for *M. abscessus* 16S rRNA	gtaatgccggggactcgtag
Mab_16S_rRNA_R	Reverse qPCR primer for *M. abscessus* 16S rRNA	tagcatgtgtgaagccctgg
Msmeg_16S_rRNA_F	Forward qPCR primer for *M. smegmatis* 16S rRNA	ggcagcagtggggaatattg
Msmeg_16S_rRNA_R	Reverse qPCR primer for *M. smegmatis* 16S rRNA	gcccagtaattccggacaac
G2 PrimFor MAB_2736c	Forward primer to amplify *MAB_2736c* for insertion into pL5 PTetO Msm PonA1 truncation A-FLAG clone 1	GCATGCTTAATTAAGGAGGAGATATACATATGATTACCGCGACGGACCT
G2 PrimRev MAB_2736c	Reverse primer to amplify *MAB_2736c* for insertion into pL5 PTetO Msm PonA1 truncation A-FLAG clone 1	AAGCTTCTAGTGGTGGTGGTGGTGGTGGGCCAGCTCGATGAGGTC
sgCtrl-1top	Non-targeting sgRNA top strand	GGGAGGTCTAGTATCTTGAGCGTT
sgCtrl-1bottom	Non-targeting sgRNA bottom strand	AAACAACGCTCAAGATACTAGACC
sg2736c_1top	sgRNA targeting MAB_2736c top strand - 1	GGGAAGTTGTTGGTCGGCTCGTCGA
sg2736c_1bottom	sgRNA targeting MAB_2736c bottom strand - 1	AAACTCGACGAGCCGACCAACAACT
sg2736c_2top	sgRNA targeting MAB_2736c top strand - 2	GGGAACCAGCCCGCCATCGTGATTTT
sg2736c_2bottom	sgRNA targeting MAB_2736c bottom strand - 2	AAACAAAATCACGATGGCGGGCTGGT
sg2736c_3top	sgRNA targeting MAB_2736c top strand - 3	GGGAAAAGCGTCCGCAGCGGTTGAA
sg2736c_3bottom	sgRNA targeting MAB_2736c bottom strand - 3	AAACTTCAACCGCTGCGGACGCTTT
Ggguide_1 For	Forward primer to amplify sgRNA with appropriate SapI sites to generate triple CRISPRi plasmids - 1	AATATGCTCTTCAGGATCTGACCAGGGAAAATAGCC
Ggguide_2 Rev	Reverse primer to amplify sgRNA with appropriate SapI sites to generate triple CRISPRi plasmids - 1	TTTATGCTCTTCAGTCAAAATAAAAAAGGGGACCTCTAGGG
Ggguide_2 For	Forward primer to amplify sgRNA with appropriate SapI sites to generate triple CRISPRi plasmids - 2	AATATGCTCTTCAGACTCTGACCAGGGAAAATAGCC
Ggguide_2 Rev	Reverse primer to amplify sgRNA with appropriate SapI sites to generate triple CRISPRi plasmids - 2	TTTATGCTCTTCACTGAAAATAAAAAAGGGGACCTCTAGGG
MAB_2736c For	Forward primer to amplify *MAB_2736c* for insertion into PURExpress DHFR Control Plasmid	ATGATTACCGCGACGGACC
MAB_2736c Rev	Reverse primer to amplify *MAB_2736c* for insertion into PURExpress DHFR Control Plasmid	TTAGGCCAGCTCGATGAGGTC
mNeonGreen F	Forward primer to amplify *mNeonGreen* for insertion into PURExpress DHFR Control Plasmid	GTGAGCAAGGGCGAGGAGG
mNeonGreen R	Reverse primer to amplify *mNeonGreen* for insertion into PURExpress DHFR Control Plasmid	TTACTTGTACAGCTCGTCCATGCCC

### Mycobacterial culturing conditions

All *M. abscessus* and *M. smegmatis* cultures were grown at 37°C in Middlebrook 7H9 broth (271,310, BD Diagnostics) with 0.2% (vol/vol) glycerol (GX0185, Supelco), 0.05% (vol/vol) Tween-80 (P1754, MilliporeSigma), and 10% (vol/vol) oleic acid-albumin-dextrose-catalase (OADC) (90000-614, VWR) (*M. abscessus*) or albumin dextrose catalase (ADC) (*M. smegmatis*) with appropriate antibiotics.

### Mycobacterial transformations

*M. abscessus* or *M. smegmatis* were grown to an optical density (OD_600_) of 0.8, then washed thrice with sterile 10% glycerol by pelleting at 5,000 × *g* for 7 min at 22°C. After the final wash, the cells were resuspended in 1% of the initial culture volume in 10% glycerol; 50 µL of electrocompetent mycobacteria were mixed well with 100 ng DNA in 2 µL water and then transferred to a 2 mm electroporation cuvette (89047-208, VWR). The cells were electroporated at 2,500 V, 125 Ω, 25 μF using an ECM 630 electroporator (45-0651, BTX). In total, 1 mL 7H9 + OADC broth was added to the electroporated cells, and cells recovered for 4 h shaking at 150 rpm at 37°C; 100 µL of recovered cells were spread on 7H10 + 0.5% (vol/vol) glycerol + 10% (vol/vol) OADC agar plates with 50 µg mL^−1^ kanamycin sulfate using 4 mm borosilicate glass beads. Plates were incubated at 37°C for 4 days. For *M. smegmatis* strains, a similar method was used, but with a shortened recovery time of 2 h after electroporation, and plates were incubated for only 3 days.

### Generation of mutant MAB_2736c strains

CRISPRi MAB_2736c plasmid was constructed using Addgene plasmid 166,886 as previously described (33). Briefly, plasmid 166,886 was digested with BsmBI-v2 (NEB R0739L) and then gel-purified. Three sgRNAs were designed to target three different locations of the non-template strand of MAB_2736c. Each individual sgRNA with appropriate overhangs was annealed and ligated (T4 ligase NEB M0202M) into three separate BsmBI-v2 digested backbones. To generate a plasmid with all three sgRNAs, a SapI-based Golden Gate cloning site 3′ to the first sgRNA scaffold was used as previously described (33). Successful plasmid construction was verified using long-read sequencing (Plasmidsaurus). Non-targeting (NT) control was constructed in a similar manner but with scrambled, non-targeting sgRNAs. Triple MAB_2736c or NT plasmids were transformed into ATCC19977 and selected on 7H10 + 0.5% (vol/vol) glycerol + 10% (vol/vol) OADC agar plates containing 50 µg mL^−1^ kanamycin sulfate.

MAB_2736c constitutive plasmid was constructed into pL5 PTetO Msm PonA1 truncation A-FLAG clone 1 (40) using NdeI (R0111, NEB) and HindIII-HF (R3104, NEB) restriction digest, followed by isothermal assembly with Phusion High-Fidelity Polymerase (M0530, NEB) and transformed into ATCC19977 and *M. smegmatis*. Successful transformants were selected on 7H10 + 0.5% (vol/vol) glycerol + 10% (vol/vol) OADC agar plates containing 50 µg mL^−1^ kanamycin sulfate.

### Minimum inhibitory concentration determination

*M. abscessus* and *M. smegmatis* were grown until mid-log phase (OD_600_ of 0.6−0.8). For CRISPRi experiments, cultures were induced for knockdown 18−24 h prior to the start of the assay with 500 ng μL^−1^ ATc in DMSO. Cultures were then diluted to an OD_600_ of 0.003, and 200 μL aliquots were plated in technical triplicate in wells (3370, Corning) containing specified antibiotics or 500 ng μL^−1^ ATc when relevant. Antibiotic stocks were made as follows: 20 mg mL^−1^ linezolid (PZ0014, Sigma-Aldrich) in DMSO, 10 mg mL^−1^ clarithromycin (C9742, Sigma-Aldrich) in DMSO, 10 mg mL^−1^ amikacin disulfate salt (A1774, Sigma-Aldrich) in water, 10 mg mL^−1^ ofloxacin (O8757, Sigma-Aldrich), 1 mg mL^−1^ imipenem monohydrate (I0160, Sigma-Aldrich) in water, 20 mg mL^−1^ chloramphenicol (C0378, Sigma Aldrich) in ethanol, or 10 mg mL^−1^ tigecycline hydrate (PZ0021, Sigma Aldrich) in DMSO. The cells were then incubated at 37°C with shaking at 150 r.p.m. for 24 h; 0.002% resazurin (R7017, Sigma Aldrich) in ddH2O was then added to each well, and plates were incubated for an additional 24 h at 37°C with shaking at 150 r.p.m. MIC determination was conducted using a Tecan Spark 10M plate reader (Mannedorf, Switzerland) by measuring absorbance at 570 nm and 600 nm and normalizing the ratio to background and no drug control. Nonlinear regression was used to fit a sigmoid curve to the dose-response data, and the half-maximal minimum inhibitory concentration (MIC_50_) was calculated where applicable using GraphPad Prism (version 10.5.0). For Fig. 2D, nonlinear regression could not be fitted due to the absence of a sigmoidal dose-response relationship. In this instance, the MIC was determined by visual inspection, following the principles of the traditional resazurin microdilution assay, wherein the MIC is defined as the lowest drug concentration resulting in complete inhibition of growth (40). This approach was feasible because the unambiguous transition between growth and no growth yielded a defined cutoff concentration.

### Growth curve

CRISPRi strains were grown until mid-log phase (OD_600_ of 0.6−0.8) and pre-depleted with ATc at 500 ng mL^−1^ for 18−24 h. Cultures were then back-diluted at a final OD_600_ of 0.02, and 200 µL of diluted cells were added in technical triplicate with DMSO or linezolid at 0.5 µg mL^-^1 and fresh ATc at 500 ng mL^−1^. Growth was determined by continuous OD_600_ measurement in 15-min intervals in a Spark 10M plate reader for 48 h at 37°C with continuous shaking at 1,000 rpm. Growth curve data were analyzed using Microsoft Excel 365 and GraphPad Prism 9.

### Protein structure alignments

Tertiary protein structures for MAB_2736c from *M. abscessus* ATCC19977 and OcrA from *M. tuberculosis* H37Rv (29, 30) were predicted using AlphaFold (31). MAB_2736c predicted protein structure was aligned with the predicted structure of OcrA and the X-ray crystal structure of PoxtA from *E. faecalis* (14) using PyMOL 3.0 (41). RMSD scores were determined using the alignment function within PyMOL 3.0.

### Quantitative PCR

For the *MAB_2736c* CRISPRi strain, cultures were grown in biological triplicate to mid-log phase, diluted back in +/− 500 ng/mL ATc, and grown for 18–24 h to achieve target knockdown. For the *MAB_2736c* overexpression strains in *M. abscessus* and *M. smegmatis*, cultures were grown in biological triplicate under kanamycin selection to an OD_600_ of 0.6–0.8. 2 OD_600_ equivalents of cells from each culture were harvested by centrifugation, resuspended in TRIzol (Thermo Fisher), and lysed by bead beating (Lysing Matrix B, MP Biomedicals) 4 × 4,000 rpm. Total RNA was extracted from the TRIzol aqueous phase using column-based purification (R1018, Zymo Research). Residual genomic DNA was digested by the addition of TURBO DNase buffer (AM2238, Thermo Fisher Scientific) (final concentration of 10%) and TURBO DNase (final concentration of 2%) for 1 h at 37°C, then the samples were purified with RNA clean-up columns (R1018, Zymo Research). cDNA was prepared using random hexamers (Life Technologies Superscript IV), column-purified (28,106, Qiagen), and then quantified by RT-qPCR on a QuantStudio 7 Flex real-time PCR machine (Thermo Fisher Scientific) using iTaq Universal SYBR Green Supermix (BioRad). All qPCR primer pairs were confirmed to display a linear response to cDNA concentration over a 64-fold range covering the experimental values. Relative cDNA abundance was calculated using the ΔΔCt method through normalization to the –ATc condition and to expression of the 16S rRNA.

### Sequence variation analysis

A total of six *M. abscessus* clinical isolates with varying linezolid MIC values were included. Whole-genome sequencing, assembly, and subspecies assignment were performed previously, and genome assemblies were obtained from that study (36). The ATCC 19977 reference strain genome was obtained from Mycobrowser (30). The loci *rrl* (MAB_r5052), *rplC* (MAB_3820c), *rplD* (MAB_3819c), *MAB_2736c,* and the upstream promoter region of *MAB_2736c* were investigated in each strain. For promoter analysis, the upstream region of MAB_2736c was defined as up to 300 bp upstream of the annotated start codon. Transcriptional start site (TSS) information was derived from a previously published transcriptome atlas (42). The primary TSS was excluded because its indicated promoter was located more than 1 Mb upstream of the coding sequence. The secondary TSS located 170 bp upstream of the start codon was used for promoter definition. Annotated coding sequences and upstream sequences were extracted from gene FASTA files and protein coding genes (*rplC*, *rplD*, and *MAB_2736c*) translated prior to alignment. The −10 promoter motif was defined according to the mycobacterial TANNNT consensus previously described (43). Nucleotide and amino acid sequence alignments were performed using the MAFFT algorithm on default settings as implemented in Jalview (44, 45). Sequence variation was assessed relative to the ATCC 19977 reference strain. Variants were recorded descriptively and evaluated for correspondence with linezolid MIC values. No statistical testing was performed due to the limited sample size.

### PURExpress *in vitro* protein synthesis

Plasmids carrying MAB_2736c or mNeonGreen compatible with the PURExpress *in vitro* protein synthesis kit were cloned into the manufacturer’s PURExpress DHFR Control Plasmid (E6800L, NEB) by swapping out DHFR with MAB_2736c (PCR amplified from ATC19977 gDNA) or mNeonGreen using standard restriction cloning with NdeI (R0111S, NEB) and PacI (R0527S, NEB). PURExpress reactions were set up following the manufacturer’s protocol with either no template, 200 ng pDHFR, or 200 ng pMAB_2736c, as well as 200 ng of mNeonGreen included in every reaction. Mixed reactions were aliquoted into black bottom 384-well plates (CLS3573, Corning), and the plates were incubated at 37°C for 30 min; 100 µM, 50 µM, and 25 µM linezolid or DMSO control were spiked into appropriate wells. Plates were incubated for an additional 2 h at 37°C in Spark 10M plate reader with ex/em of 506 nm/517 nm read at 15-min intervals. PURExpress data were analyzed using Microsoft Excel 365 and GraphPad Prism 9.

### Statistical methods

For all data points, error bars represent the standard deviation of the y-variable on the graph. Statistical significance between two independent groups was queried with unpaired, two-tailed *t*-tests. For analyses involving multiple groups and experimental factors, statistical significance was assessed using two-way ANOVA followed by Šídák’s multiple comparisons test to adjust for multiple hypothesis testing. All statistical tests used are indicated in the figure legends.

### Visualization

Schematic visualizations of experiments were created with Inkscape (version 1.4.2; Inkscape Project, https://inkscape.org). Illustrative elements were adapted from Servier Medical Art, which is licensed under a Creative Commons Attribution 4.0 International License (CC BY 4.0) (https://smart.servier.com). Statistical figures were generated with GraphPad Prism 9.

## Data Availability

Genome assemblies and annotation files used in this study are publicly available from the institutional repository at https://orca2.tamu.edu/Mab_TnSeq/. These data sets were previously published (36).

## SUPPLEMENTAL MATERIAL

Figure S1 aac.01605-25-s0001.eps Sequence alignment of the *MAB_2736c* promoter region.

Supplemental legend aac.01605-25-s0002.docx Descriptive caption for Fig. S1.

ASM does not own the copyrights to Supplemental Material that may be linked to, or accessed through, an article. The authors have granted ASM a non-exclusive, world-wide license to publish the Supplemental Material files. Please contact the corresponding author directly for reuse.
