# Supplementary material for: Ribosomal protection as a linezolid resistance mechanism in Mycobacterium abscessus
Source: Antimicrob Agents Chemother. 2026 Mar 2;70(4):e01605-25. doi: 10.1128/aac.01605-25 (PMC13041422; doi:10.1128/aac.01605-25)
Supplement: Supplemental legend — Descriptive caption for Fig. S1. [file aac.01605-25-s0002.docx]

**Supplementary Figure S1. Sequence alignment of the *MAB_2736c* promoter region.**

Multiple sequence alignment of the 300 bp upstream regions relative to the *MAB_2736c* start codon from the ATCC19977 reference strain and six clinical isolates (BWH-F, Taiwan-36, Taiwan-38, Taiwan-44, Taiwan-48, Taiwan-54). Translational start site (TSS) highlighted in green, the predicted−10 promoter motif alignment in grey. Nucleotide coloring reflects sequence identity across strains.
